# Characterization and comparison of human glioblastoma models

**DOI:** 10.1186/s12885-022-09910-9

**Published:** 2022-08-03

**Authors:** Julia A. Schulz, Louis T. Rodgers, Richard J. Kryscio, Anika M.S. Hartz, Björn Bauer

**Affiliations:** 1 Department of Pharmaceutical Sciences, College of Pharmacy University of Kentucky, Lexington, KY USA; 2 Sanders-Brown Center on Aging, University of Kentucky, Lexington, USA; 3 Statistics, College of Arts and Sciences, University of Kentucky, Lexington, KY USA; 4 Pharmacology and Nutritional Sciences, College of Medicine, University of Kentucky, Lexington, USA; 5 Drug Discovery, Delivery and Translational Therapeutics Track, Markey Cancer Center, College of Medicine, University of Kentucky, Lexington, USA

**Keywords:** Glioblastoma, U87-luc2, U251-RedFLuc, Invasiveness, ABC transporters

## Abstract

Glioblastoma (GBM) is one of the deadliest cancers. Treatment options are limited, and median patient survival is only several months. Translation of new therapies is hindered by a lack of GBM models that fully recapitulate disease heterogeneity. Here, we characterize two human GBM models (U87-luc2, U251-RedFLuc). In vitro, both cell lines express similar levels of luciferase and show comparable sensitivity to temozolomide and lapatinib exposure. In vivo, however, the two GBM models recapitulate different aspects of the disease. U87-luc2 cells quickly grow into large, well-demarcated tumors; U251-RedFLuc cells form small, highly invasive tumors. Using a new method to assess GBM invasiveness based on detecting tumor-specific anti-luciferase staining in brain slices, we found that U251-RedFLuc cells are more invasive than U87-luc2 cells. Lastly, we determined expression levels of ABC transporters in both models. Our findings indicate that U87-luc2 and U251-RedFLuc GBM models recapitulate different aspects of GBM heterogeneity that need to be considered in preclinical research.

**Supplementary Information:**

The online version contains supplementary material available at 10.1186/s12885-022-09910-9.

## Summary statement

We compare the human GBM models U87-luc2 and U251-RedFLuc based on tumor growth, invasiveness, drug resistance, and survival. Both models recapitulate GBM heterogeneity which is critical in preclinical research.

## Introduction

Glioblastoma (GBM) is the most common primary brain tumor and among the most devastating human diseases (Ostrom et al. [38]). In the US, the annual incidence of GBM is 3.23 per 100,000 people (Ostrom et al. [38]). Due to frequent recurrences within 6–9 months after resection, most GBM patients survive less than one year, and median survival of GBM patients is only 8 months after diagnosis (Mallick et al. [36]; Ostrom et al. [38]; Roy et al. [46]).

The majority of new therapeutic approaches fail to improve GBM patient survival due to low anticancer drug brain uptake, which is restricted by ABC efflux transporters at the blood-brain barrier (de Gooijer et al. [10]; Lin et al. [35]). Additionally, modeling GBM in preclinical studies is difficult. Many GBM models do not fully recapitulate the disease, including growth patterns, invasiveness, and genetic heterogeneity, and lack proper characterization and description in the literature (Ellis et al. [14]; Fomchenko and Holland [18]). Here, we characterize and compare two human GBM models, the U87-luc2 and U251-RedFLuc models, in detail.

The U87 model was established in 1968 by Ponten et al. at the University of Uppsala, Sweden, and is the most used glioblastoma model (Ponten and Macintyre [42]). U87 cells form large tumors that are highly vascularized and rarely necrotic in their core (de Vries [11]; de Vries et al. [12]; Radaelli et al. [43]). U87 cells also recapitulate the most common genetic profile of glioblastoma in that they express mutant PTEN, PI3K, and Akt (Fueyo et al. [19]; Ishii et al. [27]; Koul et al. [30]; Radaelli et al. [43]). Additionally, mutations in cell cycle control have been observed, leading to a deletion of the regulatory subunits p14 and p16 of cyclin-dependent kinases (Fueyo et al. [19]; Ishii et al. [27]). Even though U87 cells are of glial origin, they do not express two common astrocytic markers, GFAP and S100β. However, GBM can present with GFAP staining around the borders due to infiltration of reactive astrocytes (Candolfi et al. [8]; Radaelli et al. [43]).

In 1973, Bengt Westermark’s laboratory at the University of Uppsala established the U251-MG cell line, the second most used GBM cell line, from a 75-year-old male GBM patient (Westermark et al. [58]). Unlike the U87 model, U251 cells recapitulate many of the main glioblastoma characteristics. U251 tumors from mouse GBM models display a highly infiltrative and invasive growth pattern with small central tumor cores, edema, hemorrhage, and areas of vascular proliferation (Candolfi et al. [8]; Radaelli et al. [43]). Additionally, U251 tumors cover a broad spectrum of genetic variability, including mutant PTEN, upregulation of PI3K and Akt, non-functional p53, and aberrant expression of other proteins involved in cell cycle control (Ishii et al. [27]; Radaelli et al. [43]). Due to their astrocytic origin, U251 cells and tumors stain positive for the astrocytic markers GFAP, S100β, and vimentin (Radaelli et al. [43]).

The main advantage of human GBM models is also their main disadvantage: The tumors are of human origin, allowing direct translation of target engagement and dissection of tumor-specific signaling in mouse models. However, due to their human nature, these tumors only grow in immunocompromised mice. Therefore, these particular models cannot be used to study immunotherapies or the role of the natural tumor microenvironment in tumor progression and treatment (Ellis et al. [14]; Fomchenko and Holland [18]). Like many other human tumor cell lines, U87 and U251 cells were established several decades ago, which increases the possibility of changes in genetic and biological features due to long-term culture (Gstraunthaler [21]; Gu et al. [22]). Furthermore, the decades-long use of these cells may have resulted in contamination or mix-up in repositories, which complicates authenticating these cell lines (Allen et al. [1]; Stepanenko and Kavsan [48]). In the present study, the information discussed and cited refers to the ATCC U87-MG (HTB-14) and ECACC U251-MG (09063001) cell lines.

Nevertheless, the U87 and U251 GBM models are commonly used and have many well-described features. Here, we characterize and directly compare the U87-luc2 and U251-RedFLuc GBM models and evaluate several in vitro and in vivo endpoints, including luciferase expression and activity, tumor growth, volume and invasiveness, survival, and ABC transporter expression.

## Materials  and methods

### Chemicals

Lapatinib and temozolomide were purchased from Selleckchem (Houston, TX, USA). Antibodies against P-gp (ab170904, RRID:AB_2687930), BCRP (BXP-53, ab24115, RRID:AB_447879), β-actin (ab8226, RRID:AB_306371), MRP1 (ab260038, RRID:AB_2889834), MRP4 (ab77184, RRID:AB_1523967) and firefly luciferase (ab187340, RRID:AB_2889836 (DB) and ab181640, RRID:AB_2889835 (IHC)), as well as recombinant firefly luciferase protein (ab100961), were obtained from Abcam (Cambridge, MA, USA). Antibody against P-gp (C219, 517,310, RRID:AB_564389) was purchased from MilliporeSigma (St. Louis, MO, USA). Horseradish peroxidase-conjugated secondary antibodies against rat (31,470, RRID:AB_228356), mouse (31,430, RRID:AB_228307), goat (31,402, RRID:AB_228395) and rabbit (31,460, RRID:AB_228341) IgG were purchased from Thermo Fischer Scientific (Waltham, MA, USA). PBS and DPBS were purchased from HyClone (Logan, UT, USA), and DMSO was acquired from MilliporeSigma (St. Louis, MO, USA).

### Animals

 All animal experiments were approved by the University of Kentucky Institutional Animal Care and Use Committee (protocols 2015–2168 and 2018–2947; PI: Bauer) and were carried out per AAALAC regulations, the US Department of Agriculture Animal Welfare Act, and the Guide for the Care and Use of Laboratory Animals of the NIH.

Male homozygous J:NU mice (Stock No. 007850; Jackson Laboratories, Bar Harbor, ME, USA) were delivered at age 5 weeks with an average body weight of 23.3 ± 0.5 g (SEM). Animals were group-housed in an AAALAC-accredited temperature- and humidity-controlled barrier facility (21–22 °C, 30–70% relative humidity, 14:10 light-dark cycle) with an EcoFlo Allentown ventilation system (Allentown Inc., Allentown, NJ, USA). Mice had *ad libitum* access to tap water and standard rodent feed (Envigo Teklad Chow 2918, Envigo, Indianapolis, IN, USA). After arrival, animals were allowed to habituate to the vivarium for at least one week before they were used for experiments.

### Cell culture

*Human Bioware® Brite U87-luc2 cells *were purchased from PerkinElmer (BW124577, RRID:CVCL_5J12, PerkinElmer, Waltham, MA, USA). U87-luc2 cells were cultured in Minimum Essential Medium (MEM) containing 1.5 g/l sodium bicarbonate, NEAA, L-glutamine, and sodium pyruvate (10-009-CV, Corning, Corning, NY, USA) with 10% fetal bovine serum (FBS; 89510-186, VWR, Radnor, PA, USA) and 2 µg/ml puromycin (1861 − 100, BioVision, Milpitas, CA, USA) at 37 °C, 5% CO_2_. Proliferation and appearance of cell cultures were assessed with a Telaval 31 light microscope (100x magnification; 5,501,470, Zeiss, White Plains, NY, USA). Confluency was visually estimated by evaluating cell coverage of the flask bottom under the microscope. At approximately 95% confluence, U87-luc2 cells were trypsinized (0.05% trypsin, 5 ml; 25-053-CI, Corning, Corning, NY, USA) for 5 min in the incubator. Trypsinization was stopped by adding 10 ml medium containing 10% FBS. After centrifugation (100 g, 5 min, room temperature), cells were resuspended in medium, counted with a Scepter 2.0 Handheld Automated Cell Counter (MilliporeSigma, St. Louis, MO, USA), and used for experiments.

*Human U251-MG cells* were purchased from MilliporeSigma (09063001-1VL, RRID:CVCL_0021, St. Louis, MO, USA) and cultured in MEM with 1.5 g/l sodium bicarbonate, NEAA, L-glutamine, and sodium pyruvate (10-009-CV, Corning, Corning, NY, USA) containing 10% FBS (89510-186, VWR, Radnor, PA, USA) and 2X penicillin/streptomycin (15140-122, Life Technologies, Carlsbad, CA, USA) at 37 °C, 5% CO_2_. Proliferation and appearance of cell cultures were assessed with a Telaval 31 light microscope (100x magnification; 5,501,470, Zeiss, White Plains, NY, USA). For experiments, U251-MG cells were trypsinized as described above.

*Human U251-RedFLuc cells* were generated by transducing U251-MG cells using the RediFect™ Red-FLuc-Puromycin Lentiviral Particles kit (CLS960002, PerkinElmer, Waltham, MA, USA) according to the manufacturer’s instructions. Briefly, 50,000 cells/well were seeded in a 24-well plate (Costar, 3738, Corning, NY, USA) and grown in antibiotic-free MEM supplemented with 10% FBS at 37 °C, 5% CO_2_. After 24 h, the medium was replaced with 1 ml medium containing 4 µg/ml polybrene (MilliporeSigma, St. Louis, MO, USA). Lentiviral particles were added at a multiplicity of infection of 20, and cells were incubated at 37 °C, 5% CO_2_. After 24 h, the medium was replaced with penicillin/streptomycin-containing medium, and cells were cultured for another 48 h. On day 3, the medium was replaced with puromycin-containing medium to select FLuc-expressing cells. After this selection, cells were cultured in MEM with sodium bicarbonate, NEAA, L-glutamine, and sodium pyruvate (Corning, Corning, NY, USA) containing 10% FBS (VWR, Radnor, PA, USA) and 2 µg/ml puromycin (BioVision, Milpitas, CA, USA) at 37 °C, 5% CO_2_. Successful transduction was confirmed with in vitro bioluminescence imaging, as described below. Proliferation and appearance of cell cultures were assessed with a Telaval 31 light microscope (100x magnification; 5,501,470, Zeiss, White Plains, NY, USA). For experiments, U251-RedFLuc cells were trypsinized as described above.

### *In Vitro* bioluminescence imaging

We confirmed luciferase expression and activity in transduced cells with in vitro bioluminescence imaging. 10,000, 15,000, and 20,000 GBM cells/well were plated in black clear-bottom 96-well plates (Corning, Corning, NY, USA). The medium was replaced with 100 µl DPBS supplemented with 5 mM D-glucose and 1 mM sodium pyruvate (MilliporeSigma, St. Louis, MO) after 24 h. XenoLight® RediJect™ D-Luciferin (150 µg/ml; PerkinElmer, Waltham, MA, USA) was diluted 1:100 in DPBS supplemented with 5 mM D-glucose and 1 mM sodium pyruvate. Immediately after adding 100 µl of XenoLight® RediJect™ D-Luciferin dilution to each well (1:100 dilution, 0.15 µg/well), bioluminescence images were acquired with an IVIS® Spectrum (FOV: 13 cm, f-stop: 1, binning: 4; 124,262, PerkinElmer, Waltham, MA, USA). Bioluminescence was recorded every minute for 5 min, then every 5 min for 25 min. Bioluminescence was analyzed with Living Image 4.7.3 (PerkinElmer, Waltham, MA, USA) using a 96-well grid ROI. Bioluminescence for each well was expressed as total photons/s.

### MTT cytotoxicity assay

For experiments with temozolomide, 3,000 cells/well were plated into 96-well plates (Costar, 3599, Corning, NY, USA). After 24 h, cells were exposed to temozolomide (0-500 µM in MEM with 10% FBS and 2 µg/ml puromycin) for 24, 48 h, or 72 h. For experiments with lapatinib, 10,000 cells/well were plated into 96-well plates (Costar, 3599, Corning, NY, USA). After 24 h, cells were exposed to lapatinib (0-500 µM in MEM with 10% FBS and 2 µg/ml puromycin) for 24, 48 h, or 72 h. Drug stock solutions were prepared in DMSO, and further diluted in MEM supplemented with 10% FBS and 2 µg/ml puromycin.

According to the manufacturer’s instructions, cell viability was assessed with the Vybrant® MTT Cell Proliferation Assay Kit (Thermo Fisher, Waltham, MA, USA). MTT was dissolved in PBS to a 12 mM stock solution that was further diluted 1:1,000 in the phenol red-free MEM (Gibco, Thermo Fisher Scientific, Waltham, MA, USA) with 10% FBS. 100 µl MTT solution (diluted to 12 µM) was added per well, and cells were incubated for 4 h at 37 °C, 5% CO_2_. SDS was dissolved in 0.01 M HCl to a final concentration of 0.3 M and added to each well. Then, the plate was shaken for 4 h at 37 °C. Formazan absorbance was read at 570 nm with a Synergy H1 plate reader (BioTek, Winooski, VT, USA). After subtracting the blank values, cell viability was reported as % of control. IC50 values were determined in GraphPad Prism® (version 9) using the [Inhibitor] vs. normalized response, least squares fit function.

### GBM cell implantation

GBM cells were implanted orthotopically into the brains of mice based on a previously published protocol from Carlson et al. [9]). On the day of the procedure, mice were injected with Buprenorphine HCl SR Lab (1 mg/kg, s.c.; Zoopharm, Laramie, WY, USA).

GBM cells were collected by trypsinization as described above and resuspended in sterile PBS (HyClone, Logan, UT, USA; 1.05 mM KH_2_PO_4_, 154.0 mM NaCl, 5.6 mM Na_2_HPO_4_) at the appropriate dilutions (Table S1). Cell suspensions were kept on ice during the entire implantation procedure. Mice were anesthetized with isoflurane (induction: 3%, maintenance: 0.5-2%, room air: 21% O_2_) using a SomnoSuite Rodent Anesthesia machine (Kent Scientific, Torrington, CT, USA) and positioned in a stereotaxic frame (David Kopf Instruments, Tujunga, CA, USA) on a platform with an infrared heating pad. Eyes were lubricated with OptixCare® Eye Lube (Covetrus, Portland, ME, USA), and the dorsal side of the head was disinfected by alternated swabbing with chlorhexidine (Vetoquinol, France) and sterile saline (Covetrus, Portland, ME, USA) three times for 1 min each. A 1 cm midline incision was made with a 10-blade disposable scalpel (VWR, Radnor, PA, USA), and the skin was pulled back with skin retractors (17000-01, Fine Science Tools, Foster City, CA, USA) to expose the skull. The skull was swabbed with 3% H_2_O_2_ (VWR, Radnor, PA, USA) and 70% Ethanol (VWR, Radnor, PA, USA) until bregma was visible. The injection site was localized 3 mm to the right and 1 mm anterior of bregma (primary somatosensory cortex, forelimb, and jaw regions), and a hole was drilled through the skull using a Dremel drill (tip diameter: 0.9 mm; Fine Science Tools, Foster City, CA, USA). GBM cells were mixed until homogenously suspended, and the appropriate volume of GBM cell suspension was pulled into a 5 or 10 µl Hamilton microliter syringe (Hamilton Company, Reno, NV, USA; Table S1). The needle was inserted through the drill hole and lowered 4 mm into the brain parenchyma. Then, the needle was pulled back 1 mm to create a small pocket for the cell suspension. Cell injections were performed using a UMP3T-1 UltraMicroPump 3 with SMARTouch™ Controller (David Kopf Instruments, Tujunga, CA, USA) at the appropriate injection rate (Table S1). Control mice received a PBS mock injection instead of cells. After injection, the needle remained in the brain parenchyma for 1 min before removal. The injection hole was closed with melted bone wax (Covetrus, Portland, ME, USA), and the skin was closed with wound clips (Fine Science Tools, Foster City, CA, USA). Mice were moved to preheated cages and closely monitored until they returned to the sternal position and showed normal behavior (e.g., movement, food, and water intake). Mice were monitored at least once daily throughout the entire study period until they lost more than 25% bodyweight (Toth [51]; Wallace [56]) or for a maximum of 120 days after tumor implantation.

### *In Vivo* bioluminescence imaging

To track the growth of luciferase-expressing GBM cells, mice underwent weekly in vivo bioluminescence imaging with an IVIS® Spectrum (PerkinElmer, Waltham, MA, USA) starting on day 7 after cell implantation. Mice received 5 µl/g bodyweight XenoLight® RediJect™ D-Luciferin by i.p. injection (PerkinElmer, Waltham, MA, USA). 8 min after injection, mice were anesthetized with 3.5% isoflurane and transferred to the heated imaging stage. Anesthesia was maintained with 0.5-2% isoflurane during the imaging process. Tumor bioluminescence was determined 10 min after luciferin injection by 2D imaging (FOV: 21.6 cm, f-stop: 2, binning: 4), and images were analyzed with Living Image 4.7.3 software (PerkinElmer, Waltham, MA, USA). Tumor growth was reported as fold change in bioluminescence in relation to day 7. Tumor doubling times were determined with the “Exponential Growth with log(population)” function in GraphPad Prism® (version 9).

### Magnetic Resonance Imaging (MRI)

MR imaging was performed at the University of Kentucky Magnetic Resonance Imaging and Spectroscopy Center using a 7T Bruker ClinScan, small animal, MRI scanner with a horizontal bore system (7.0 T, 30 cm, 300 Hz) equipped with a triple-axis gradient system (630 mT/m and 6300 T/m/s) and a standard 2 × 2 array surface coil. Scans were acquired 2–5 days before the point of median survival of the respective GBM model. Mice were anesthetized with 3% isoflurane, and a tail vein catheter was placed for gadolinium administration. Mice were transferred to a heated imaging coil, and anesthesia was maintained at 1–2% isoflurane; the breathing rate was continuously monitored and recorded every 10 min.

Standardized sequences for pre-gadolinium T1 (TR = 200 ms, TE = 3.1 ms, field of view (FOV): 32.00 × 16.00 × 16.00 mm), and T2-RARE (TR = 2000 ms, TE = 45 ms, RARE factor: 24, FOV: 32.00 × 16.00 × 16.00 mm) were acquired. After completing pre-contrast T1 and T2-RARE imaging, mice received i.v. gadolinium (0.6 mmol/kg; 1:10 dilution in sterile saline; 120 µl for a 20 g mouse; Gadobutrol, Gadavist™, Bayer AG, Whippany, NJ, USA) through the tail vein catheter. Post-contrast MR images (TR = 200 ms, TE = 3.1 ms, field of view (FOV): 32.00 × 16.00 × 16.00 mm) were acquired 15 min after gadolinium injection.

Image analysis was performed with *syngo*.via VB40 software (Siemens Healthineers USA, Malvern, PA, USA). The pre-contrast image was subtracted from the respective post-contrast image using the Subtraction Tool. The tumor area of the enhancing tumor A_T_ was determined for each slice, and tumor volume (V_T_) was calculated with the following equation (Eq. 1):

$${V}_{T}=\sum {A}_{T}*{h}_{T}$$

where h_T_ represents the slice thickness (450 μm).

### Histopathology

Mice with more than 25% weight loss (Toth [51]; Wallace [56]) were anesthetized with pentobarbital (FatalPlus®, 150 mg/kg, i.p.) for brain perfusion. The animals’ chest cavity was opened, the descending aorta was clamped, and a small cut was made to the right ventricle. The infusion needle was inserted into the left ventricle, and the brain was perfused with 100 ml PBS (5 ml/min) followed by 50 ml of 10% formalin (5 ml/min; 100,496; MilliporeSigma, St. Louis, MO, USA). After the perfusion, mice were decapitated, and brains were removed and incubated in 10% formalin at room temperature for 24 h. Brains were then placed in 70% ethanol and stored at 4ºC until further processing by the Biospecimen Procurement and Translational Pathology Shared Resource Facility of the University of Kentucky Markey Cancer Center, following standard protocols.

Formalin-fixed tissue was dehydrated with increasing concentrations of ethanol (70–100%), followed by de-fatting in xylene. The brain tissue was then infiltrated and embedded in paraffin and formalin-fixed, paraffin-embedded tissue was sectioned (Fischer et al. [15], Fischer et al [17]); two consecutive 4 μm slices were acquired every 200 μm throughout the tumor. After de-paraffinization with xylene and rehydration in decreasing concentrations of ethanol (100 − 70%), the first slice was used for anti-luciferase immunohistochemistry; the second slice was used for hematoxylin and eosin (H&E) staining.

Luciferase immunohistochemistry was performed on the Ventana Discovery Ultra instrument (Ventana Medical Systems, Tucson, Arizona, USA) as per the manufacturer’s instructions. In brief, brain slices were stained using CC1 standard antigen retrieval and Luciferase antibody (1 mg/ml (1:1,000 dilution), ab181640, Abcam, Cambridge, MA, USA) at 37 °C for 1 h. Slices were incubated with the OmniMap anti-Goat multimer RUO (760–4647, Ventana Medical Systems, Tucson, Arizona, USA) and with DAB chromogen according to the manufacturer’s recommendation. Slices were counterstained with hematoxylin following a standard protocol (Fischer et al. [16]; Kiernan [29]). Consecutive slices were stained with H&E using a standard protocol (Fischer et al. [16]; Kiernan [29]).

Stained brain slices were scanned with an Aperio ScanScope XT (Leica Biosystems, Buffalo Grove, IL, USA) at a 20X magnification, and images were analyzed using the Halo® image analysis platform (IndicaLabs, Albuquerque, NM, USA). The tumor area (A_T_) was determined for each IHC slice using the Halo® Multiplex IHC tool. Each IHC slice was compared to the corresponding H&E-stained slice to confirm accurate tumor localization. Tumor volume (V_T_) was calculated as noted above (Eq. 1). For histology, h_T_ represents the distance between the slices (200 μm).

The invasiveness of glioblastoma cells was analyzed in two ways. First, all IHC slices were scored based on the following parameters: invading cell edge of the main tumor, single invasive cells, or cell nests further away from the tumor. Based on the presence of these features, each slice received a score between 0 and 3. The scores for each animal were added for a total invasiveness score (Zhao et al. [63]). Second, cells outside of the main tumor area positive for both luciferase and hematoxylin staining were automatically counted in Halo^®^ using the Halo^®^ IHC Multiplex algorithm. The total number of invading cells was presented as the sum of invading cells on all slices for each animal (Lagerweij et al. [31]; Schuster et al. [47]).

### Mouse GBM samples

Mice with more than 25% weight loss were euthanized with CO_2_, followed by decapitation (Toth [51]; Wallace [56]). Mock-injected control mice for each model were euthanized at the same time. Brains were removed and divided into the contralateral and ipsilateral hemispheres. The ipsilateral hemisphere was further dissected into visible tumor and normal-appearing tissue. Samples were snap-frozen in liquid N_2_ and stored at -20ºC until further use.

### Human GBM samples

Four human GBM tissue samples were provided by the NCI Cooperative Human Tissue Network (CHTN). Note that the CHTN may have provided samples from the same tissue specimens to other investigators. Two additional human GBM tissue samples were provided by the Biospecimen Procurement and Translational Pathology Shared Resource Facility of the University of Kentucky Markey Cancer Center. Upon receipt, samples were stored at -80ºC until use.

Brain samples from six Control Individuals (CI) were obtained through the University of Kentucky Tissue Bank (IRB #B15-2602-M). Inclusion criteria were enrollment in the UK longitudinal autopsy cohort and a post-mortem interval of less than 4 h (Nelson et al. [37]). Cases with underlying CNS disorders were excluded. Due to the young age of the GBM patients, age-matched controls were not available. Patient demographics for all human samples are listed in Table S2.

### Membrane fraction and cell lysate isolation

Crude membrane fraction and lysate from cultured GBM cells as well as mouse and human tumor and control samples were isolated as described previously for isolated brain capillaries (Hartz et al. (23, 24)). Briefly, cultured GBM cells were collected by trypsinization as described above and centrifuged at 4,700 g for 1 min at 4ºC. The supernatant was removed; cells were resuspended in 1.5 ml CelLytic™ MT Cell Lysis reagent (MilliporeSigma, St. Louis, MO, USA) with 2.5X cOmplete™ Protease Inhibitor (Roche, Basel, Switzerland), and the cell suspension was transferred into ultracentrifuge tubes. Human and mouse GBM and control brain samples were thawed and transferred into tared ultracentrifuge tubes to record the tissue sample weight. CelLytic™ MT Cell Lysis reagent (MilliporeSigma, St. Louis, MO, USA) with 2.5X cOmplete™ Protease Inhibitor (Roche, Basel, Switzerland) was added at a ratio of 1:10 based on tissue sample weight. All samples (isolated tissue and cultured cells) were homogenized with a Polytron Ultraspeed Homogenizer (Probe Ø: 5 mm; 30,000 rpm for 200 s). The homogenate was centrifuged at 100,000 g for 30 min at 4ºC. Half of the supernatant was collected as cell lysate. The other half of the supernatant was transferred into fresh ultracentrifuge tubes and centrifuged at 1,000,000 g for 2 h at 4ºC. Afterward, the supernatant was discarded, and the pellet was resuspended in PBS with CelLytic™ MT Cell Lysis reagent (1:1) and cOmplete™ Protease Inhibitor (Roche, Basel, Switzerland) to collect the crude membrane fraction. Membrane fraction and cell lysate samples were frozen and stored at -20ºC.

### Western blotting

Protein expression levels of collected membrane fraction samples were determined by Western blotting using the Invitrogen NuPAGE® electrophoresis and blotting system (Carlsbad, CA, USA) as previously published (Hartz et al. [23, 24]). Before Western blotting analysis, sample protein concentrations were determined with the Bradford assay (Bradford [5]). The protein concentration of the membrane fraction samples was adjusted to 1 µg/µl (10 µg total protein content per well) for mouse samples, and 0.667 µg/µl (20 µg total protein content per well) for human samples, and samples were loaded onto the gels (NuPAGE® SDS; Invitrogen, Carlsbad, CA, USA). Gel electrophoresis was performed at a constant voltage of 200 V until complete separation of the molecular weight marker (Rainbow™, GE Healthcare Biosciences, Piscataway, NJ, USA) using a XCell Sure Lock™ Mini-Cell (Invitrogen, Carlsbad, CA, USA). After gel electrophoresis, proteins were transferred onto a PVDF membrane (Invitrogen, Carlsbad, CA) at 30 V for 2 h and blocked with T20 protein-free blocking buffer for 1 h at room temperature (Pierce Biotechnology, Rockford, IL, USA). Membranes were incubated with primary antibody (P-gp: C219, 1 µg/µl; BCRP: BXP53, 1 µg/µl; MRP1: 1 µg/µl; MRP4: 0.5 µg/µl; β-Actin, 1 µg/µl) overnight at 4 °C. Membranes were washed and incubated with secondary antibody conjugated to horseradish peroxidase for 1 h at room temperature. Following washing, membranes were incubated with SuperSignal^→^ West Pico or Femto Chemiluminescent Substrate (Pierce Biotechnology, Rockford, IL, USA). Protein bands were visualized using a BioRad Gel Doc™ XRS imaging system (BioRad, Hercules, CA, USA). Images were analyzed with ImageLab 6.1.0 (BioRad, Hercules, CA, USA).

### Dot blot

Dot blots of lysate samples from in vitro cell cultures were performed with the Whatman Minifold I 96-well system as previously described (Hartz et al. [25]). Before dot blotting, sample protein concentrations were determined with the Bradford assay (Bradford [5]). The protein concentration of the GBM cell lysate samples was adjusted to 0.01 µg/µl (2 µg total protein content per well in 200 µl). Recombinant luciferase (ab100961, Abcam, Cambridge, MA, USA) was diluted to 0.0025-0.02 ng/µl (0.5–4 ng total protein content per well). 200 µl of each solution was loaded on Amersham Protran Nitrocellulose Membranes (115–125 µg IgG/cm², GE Healthcare, Chicago, IL, USA). Membranes were processed and imaged as described in Western blotting. Luciferase protein was detected with anti-Luciferase antibody (0.25 µg/µl; ab187340, Abcam, Cambridge, MA, USA). Images were analyzed with ImageLab 6.1.0 (BioRad, Hercules, CA, USA), and the standard curve was determined with GraphPad Prism® (version 9). The protein content of the cell samples was quantified with the determined standard curve (representative standard curve: Fig. S1).

### Statistics

Data are presented as mean ± SEM. Analysis of variance (ANOVA) or a two-tailed unpaired Student’s *t-test* was used to evaluate differences between controls and treated groups using GraphPad Prism® (version 9); differences were considered to be statistically significant when *p* < 0.05. Animal survival was analyzed using the Kaplan-Meier survival function in GraphPad Prism®. Survival time was calculated as time from tumor implantation.

## Result

### Confirmation of luciferase transduction

We confirmed successful luciferase transduction of U251-MG cells by determining luciferase protein expression with dot blots and measuring luciferase activity with in vitro bioluminescence imaging. In addition, we compared these results with the commercially available U87-luc2 cells.

**Fig. 1:**
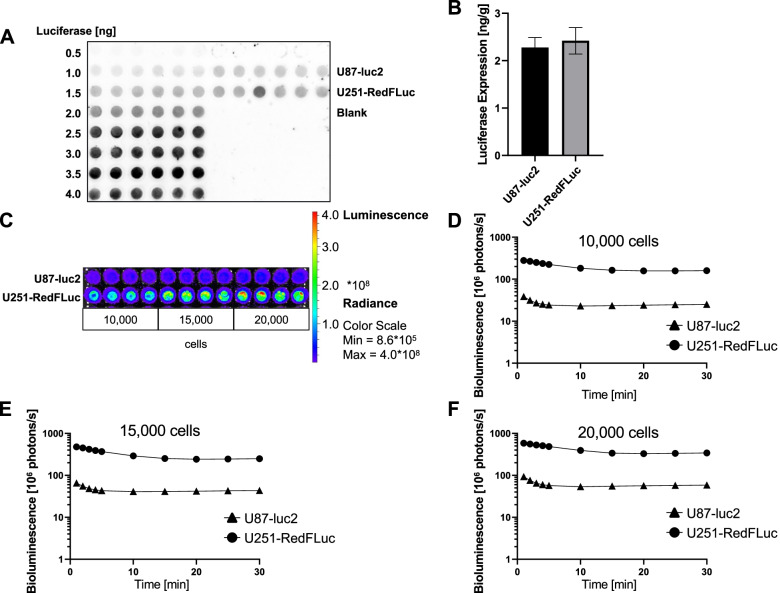
Confirmation of Luciferase Transduction *in vitro* (**A**) Representative image of a dot blot. Luciferase standard curve 0.5–4 ng. GBM cell lysate (2 µg protein loading, *n* = 6, 5 technical replicates). **B** Quantification of luciferase expression. Luciferase expression is not significantly different between U251-RedFLuc cells and U87-luc2 cell lysate samples (*p* > 0.05; *n* = 6, 5 technical replicates). **C** Representative image of in vitro bioluminescence signal of 10,000, 15,000, and 20,000 U87-luc2 or U251-RedFLuc cells 1 min after luciferin exposure (*n* = 4, 3 technical replicates). **D** In vitro bioluminescence signal of 10,000 GBM cells for U87-luc2 and U251-RedFLuc. Luciferase activity is significantly higher in U251-RedFLuc cells than U87-luc2 cells (*p* < 0.0001; *n* = 4, 3 technical replicates). **E** In vitro bioluminescence signal of 15,000 GBM cells for U87-luc2 and U251-RedFLuc. Luciferase activity is significantly higher in U251-RedFLuc cells than U87-luc2 cells (*p* < 0.0001; *n* = 4, 3 technical replicates). **F** In vitro bioluminescence signal of 20,000 GBM cells for U87-luc2 and U251-RedFLuc. Luciferase activity is significantly higher in U251-RedFLuc cells than U87-luc2 cells (*p* < 0.0001; *n* = 4, 3 technical replicates). Statistics: Unpaired *t*-test (U251-RedFLuc vs. U8-luc2; Mean ± SEM)

We detected luciferase expression and activity in both GBM cell lines, indicating successful luciferase transduction. Luciferase expression was quantified by dot blot using recombinant firefly luciferase protein as a standard. Luciferase expression levels were approximately 2 ng/g protein in cell lysate samples and not significantly different between the two cell lines (*p* < 0.69; Fig. 1A, B).

We incubated the GBM cell lines U87-luc2 and U251-RedFLuc with the luciferase substrate D-luciferin and measured bioluminescence as emitted photons/s (Fig. 1C). The bioluminescence signal was strongest 1 min after adding luciferin and decreased exponentially within the first 10 min; photon emission plateaued and remained stable for the next 20 min (Fig. 1D-F). Bioluminescence increased with increasing cell number in both GBM cell lines (Fig. 1D-F). The bioluminescence signal was significantly higher in U251-RedFLuc cells than in U87-luc2 cells for all three conditions (*p* < 0.0001). These results indicate that both GBM cell lines express active luciferase.

However, while luciferase activity of 20,000 cells after 1 min was 6-folder higher in U251-RedFLuc cells than in U87-luc2 cells, luciferase expression was not significantly different between the two cell lines. This discrepancy between luciferase expression and activity is most likely due to the differences in luciferase conversion efficacy among the constructs used for transduction of the respective cell lines. U87-luc2 cells were transduced with luc2 that is known to have a lower conversion efficacy and brightness than Red-FLuc used in the U251-RedFLuc cells (Gil et al. [20]; Liang et al. [34]; Perkin Elmer [39]).

### Cytotoxicity

GBM is a highly heterogeneous disease where cells within the same tumor can express different driver mutations and resistance mechanisms (Bai et al. [3]; Brennan et al. [7]; Kersch et al. [28]; Randall et al. [44]). To assess the drug response of each GBM cell line, we determined cell sensitivity to the standard of care drug temozolomide as well as the EGFR inhibitor lapatinib.

**Fig. 2:**
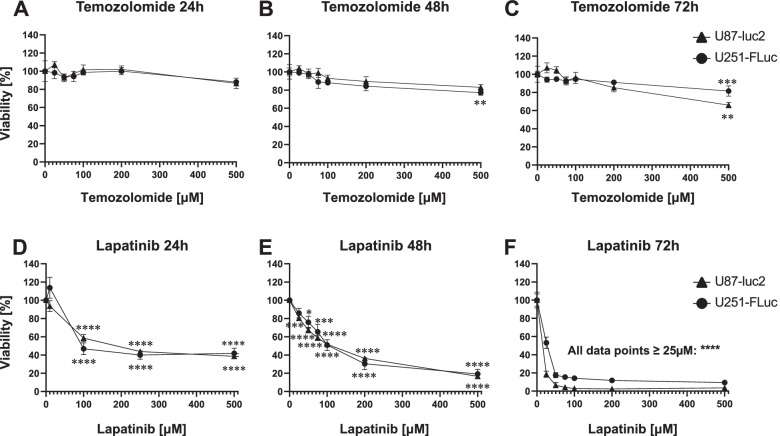
Cytotoxicity in GBM cells *in vitro***A**-**C**) After 48 h, TMZ significantly decreased the viability of U87-luc2 cells at 500µM. After 48 and 72 h, TMZ significantly decreased the viability of U251-RedFLuc cells at 500µM (*n* = 3, 3 replicates). **D**-**F** Lapatinib significantly decreased the viability of U87-luc2 and U251-RedFLuc cells at 48 and 72 h (48 h: IC_50_ = 106 µM and 119 µM; 72 h: IC_50_ = 4.78 µM and 18.8 µM, respectively; *n* = 4, 3 replicates). Statistics: Ordinary One-Way ANOVA with Dunnett’s multiple comparisons test (compared to 0 µM: *, *p* < 0.05; **, *p* < 0.01; ***, *p* < 0.001; ****, *p* < 0.0001. Unpaired *t*-test (U251-RedFLuc vs. U8-luc2; ns, *p* > 0.05; Mean ± SEM)

The cell viability of U87-luc2 was significantly decreased 72 h after exposure to 500 µM temozolomide (*p* = 0.0002; Fig. 2C). The cell viability of U251-RedFLuc cells was significantly decreased 48 and 72 h after exposure to 500 µM temozolomide (*p* = 0.0023 and *p* = 0.0028; Fig. 2B-C). Temozolomide IC_50_ values were > 500 µM for both U87-luc2 and U251-RedFLuc cells (Fig. 2 A-C, Table S1). In addition to temozolomide, we also tested the EGFR inhibitor lapatinib and its effect on cell viability in both GBM cell lines. Within 24 h, 100 µM lapatinib significantly decreased cell viability in both U87-luc2 and U251-RedFLuc cells (*p* < 0.0001 Fig. 2D). At 48 h, 50 µM lapatinib significantly decreased cell viability in U251-RedFLuc cells (U251-RedFLuc: *p* = 0.024 Fig. 2E), whereas cell viability in U87-luc2 cells was already significantly decreased at 25 µM lapatinib. (U87-luc2: *p* < 0.0001 Fig. 2E). At 72 h, 25µM significantly decreased cell viability in both cell lines (*p* < 0.0001, Fig. 2F). The IC_50_ values of lapatinib after 48 h did not significantly differ between the two GBM cell lines, indicating a consistent in vitro response to lapatinib across both GBM cell lines (Table S3).

The DMSO concentration (0.1%) used to dissolve the drugs in these experiments was not cytotoxic to either of the two GBM cell lines (U87-luc2: *p* = 0.85; U251-RedFLuc: *p* = 0.998; Fig. S2).

### *In Vivo* GBM models

#### In Vivo tumor bioluminescence

**Fig. 3:**
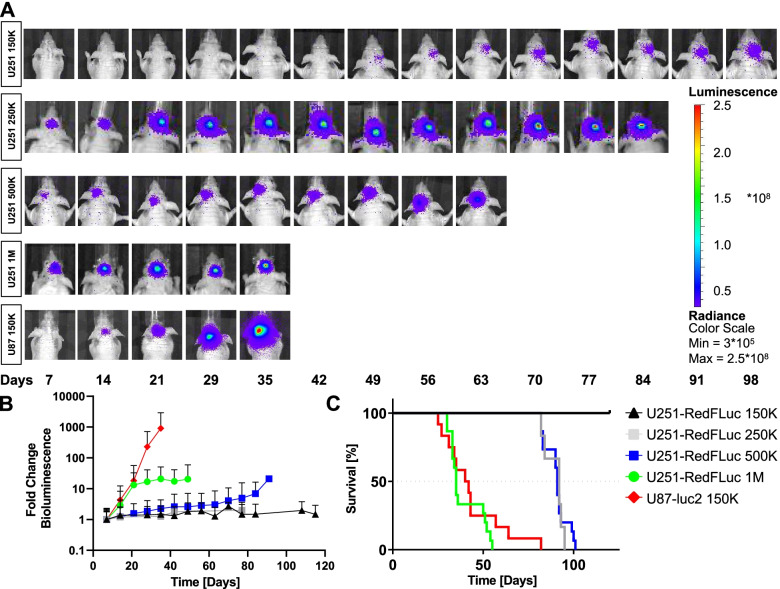
*In Vivo* Tumor Bioluminescence and Survival in Mouse GBM Models (**A**) Representative images of tumor bioluminescence in Mouse GBM Models over the entire animal lifespan. **B** Fold change in bioluminescence in mice injected with different numbers of U251-RedFLuc cells and mice injected with 150,000 U87-luc2 cells. **C** Kaplan-Meier Curves of survival of mice injected with different numbers of U251-RedFLuc cells and mice injected with 150,000 U87-luc2 cells. Statistics: One-Way ANOVA with Dunnett’s multiple comparisons test (vs. U8-luc2; **, *p* < 0.01; ****, *p* < 0.0001)

We used the GBM cell lines described above to establish mouse GBM models by orthotopically implanting the cells into the primary somatosensory cortex of immunocompromised mice. Tumor growth was monitored weekly with in vivo bioluminescence imaging of each mouse; representative images from one mouse per GBM model are shown in Fig. 3A. We detected bioluminescence in both GBM models, however, signal intensity differed between models and injected cell numbers. Tumor bioluminescence increased over time, indicating continuous tumor growth. Specifically, tumor growth in mice injected with 150,000 U87-luc2 cells was fast, with a doubling time of just under seven days (Fig. 3B; Table 1). In comparison, tumor growth in U251-RedFLuc was slower after injecting 150,000 cells. Tumors in mice injected with 150,000 U251-RedFLuc cells did not double within the 120-day study period (Fig. 3B). Injecting a larger number of cells resulted in accelerated tumor growth (Fig. 3B; Table 1) as shown for the tumor growth curves in mice injected with 250,000, 500,000, and 1,000,000 cells. In these animals, tumor doubling times were 53 (*p* = 0.067, compared to 150,000 U251-RedFLuc cells), 34 (*p* = 0.0013, compared to 150,000 U251-RedFLuc cells) and 14 days (*p* = 0.0025, compared to 150,000 U251-RedFLuc cells), respectively (Table 1). The tumor doubling time in mice injected with 1,000,000 U251-RedFLuc cells was not significantly different from mice injected with 150,000 U87-luc2 cells (*p* = 0.14), indicating that these two GBM models have comparable growth characteristics at the respective cell numbers.

**Table 1:** In Vivo tumor growth characteristics of mouse GBM models

Model	Doubling time [d]	Median survival [d]	N
U87-luc2 150 K	6.8 ± 2.7, ****	41	15
U251-RedFLuc 150 K	174.7 ± 39.4	120/ undefined, ****	6
U251-RedFLuc 250 K	52.7 ± 13.8, ns	92, **	6
U251-RedFLuc 500 K	34.1 ± 5.3, **	91, **	15
U251-RedFLuc 1 M	13.9 ± 3.2, **	35, ns	15

Statistics: Ordinary One-Way ANOVA with Dunnett’s multiple comparisons test: **, *p* < 0.01; ****, *p* < 0.0001

#### Median survival

Next, we determined median survival of the two mouse GBM models (Fig. 3 C; Table 1). Consistent with previously published literature, median survival of mice injected with 150,000 U87-luc2 cells was 41 days (Fig. 3 C; Table 1; Alphandery et al. [2]; Lee et al. [32]). In comparison, mice injected with the same number of U251-RedFLuc cells did not succumb to their tumors within the 120-day study period, and median survival could not be determined. Increasing the number of injected U251-RedFLuc cells to 250,000 or 500,000 decreased the median survival time to 92 days and 91 days, respectively (Fig. 3 C; Table 1). However, this survival was significantly longer than median survival in U87-luc2 injected mice (*p* = 0.013 and *p* = 0.016). In mice implanted with 1,000,000 U251-RedFLuc cells, median survival was 35 days (Fig. 3 C; Table 1). There was no significant difference in median survival of mice injected with 150,000 U87-luc2 cells or 1,000,000 U251-RedFLuc cells (*p* = 0.81). All further experiments were conducted using mice implanted with 150,000 U87-luc2 or 1,000,000 U251-RedFLuc cells.

#### MRI analysis and tumor volume

**Fig. 4:**
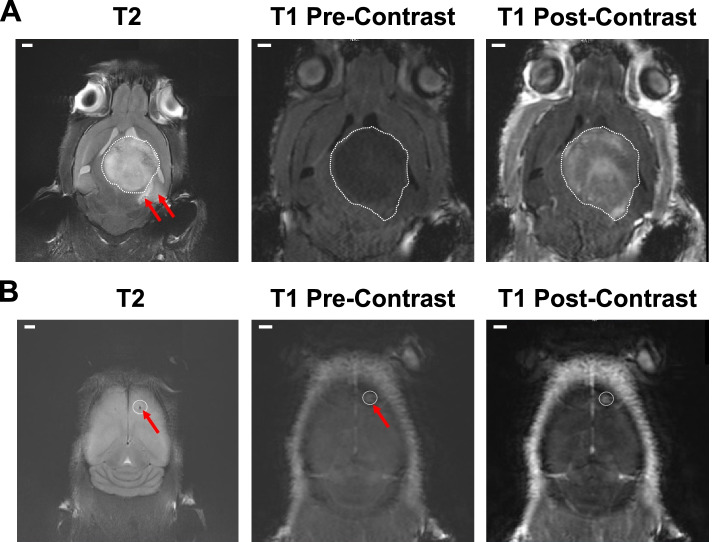
GBM Tumor Size with Gd-enhanced MRI (**A**) Representative MRI images of U87-luc2 animals. Anatomical T2 image, pre- and post-contrast T1 images. **B** Representative MRI images of U251-RedFLuc animals. Anatomical T2 image, pre- and post-contrast T1 images (*n* = 5 mice per model). White dashed line – tumor core. Red arrows – edema. Size bar = 1 mm

Tumor volume was determined with MR imaging around the time of median survival for the respective GBM model. We used a standard T2-RARE sequence to determine anatomical features and edema combined with a standard T1 sequence to evaluate contrast enhancement and determine tumor volume. Representative images of each scan for both models (150,000 U87-luc2 or 1,000,000 U251-RedFLuc cells) are shown in Fig. 4. U87-luc2 tumors appeared hyperintense on a T2-RARE anatomical scan. The large tumor cores were well demarcated and easily identifiable. Diffuse areas of edema surrounded the tumors. In most animals, the large tumors caused a midline shift and pressed into the contralateral hemisphere (Fig. 4 A). On pre-contrast T1 scans, U87-luc2 tumors appeared as hypointense, well-demarcated masses. After i.v. injection of the contrast agent gadolinium, the tumors showed heterogeneous contrast enhancement. Central areas of the tumor and some regions at the tumor rim showed stronger contrast enhancement, indicative of heterogeneous, localized blood-brain barrier disruption (Fig. 4A).

In comparison, tumors in animals injected with U251-RedFLuc cells were barely visible on the T2 scans (Fig. 4B). The injection locations were visible as small, circular, hyperintense spots in the right frontal cortex of the animals. The tumors were surrounded by areas of diffuse hyperintensity, indicating edema. The pre-contrast T1 scans showed small areas of hyperintensity in the injection location surrounded by areas of hypointensity (Fig. 4B). After contrast injection, the core region of the tumors showed significant signal enhancement.

Tumor volumes were determined based on the enhancing regions. U87-luc2 tumors were significantly larger than U251-RedFLuc tumors (Fig. 4; Table 2). The average tumor volume of U87-luc2 mice was 185 mm^3^, while the average tumor volume in U251-RedFLuc mice was 36 mm^3^ (Fig. 4; Table 2).

**Table 2:** Tumor volume

Volume [mm^3^]	MRI	Histology
U87-luc2 150 K	185 ± 43	132.2 ± 31.6
U251-RedFLuc 1 M	36 ± 9, *	96.7 ± 19.0, ns

Volume of Gd-enhancing or luciferase positive tumor for U87-luc2 and U251-RedFLuc mice (*n* = 5/group). Statistics: unpaired *t*-test *, *p* < 0.05

#### Histological analysis of tumor volume and invasiveness

**Fig. 5:**
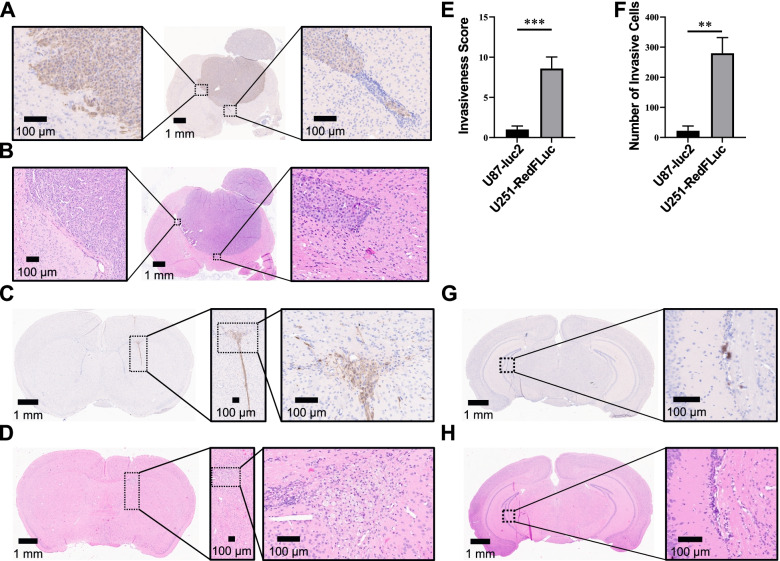
Histopathological Analysis of Tumors in Mouse GBM Models Representative images of brain slices stained for luciferase (**A**, **C**, **G**) or **H **& **E** (**B**, **D**, **H**). **A** and **B** mice implanted with U87-luc2 (150,000) cells show large central tumors with little or no invasion. **C**, **D**, **G**, **H** U251-RedFLuc (1,000,000) tumors are small and linear (**C** and **D**) with invading tumor fronts and invasive cell nests (**G** and **H**). **E** Tumor invasiveness was determined with a subjective scoring method based on anti-luciferase IHC. **F** Tumor invasiveness determined with automatic cell counting based on anti-luciferase IHC. Statistics: Unpaired *t*-test (U251-RedFLuc vs. U87-luc2) **, *p* < 0.01; ***, *p* < 0.001 (Mean ± SEM; *n* = 5 mice/model)

In H&E-stained brain slices, U87-luc2 tumors were well demarcated and easily identifiable (Fig. 5 A, B; Table 2). The tumors were large and involved both the ipsilateral and parts of the contralateral hemispheres. In some cases, tumors spread through the top of the skull and grew outside of the brain. In comparison, the main U251-RedFLuc tumors were small, linear, and only involved the ipsilateral hemispheres (Fig. 5D, Table 2). These small tumors were difficult to distinguish from healthy brain parenchyma based on H&E staining but were readily identifiable based on anti-luciferase staining (Fig. 5 C; Table 2). In addition to MRI, we also determined tumor volume based on the luciferase-positive area of the tumors. Average tumor volume in U87-luc2 mice was 132 mm^3^, which is not significantly different from the tumor volume determined with MRI (Table 2). However, average tumor volume in U251-RedFLuc mice was 97 mm^3^, which is significantly larger than the tumor volume determined with MRI (Table 2). This discrepancy between MRI and histological analysis is most likely due to the invasive nature of U251-RedFLuc tumors. Using IHC, luciferase-positive U251-RedFLuc cells were detected throughout the entire brain, indicating that tumor cells infiltrated the brain parenchyma and populated the ipsilateral and contralateral hemispheres (Fig. 5G, H). These infiltrating cells seemed to have proliferated into cell nests and small secondary tumors. While infiltrating cell nests were common in U251-RedFLuc mice, they were not detected in mice injected with U87-luc2 cells (Fig. 5G, H).

Tumor invasiveness was analyzed in two different ways. First, we employed a subjective scoring method, where each brain slice was evaluated for the presence of invading tumor front, single invasive cells, or cell nests. One or multiple of these features were present on each U251-RedFLuc brain slice, while only a few U87-luc2 brain slices showed invading tumor fronts. Based on this subjective evaluation, the U251-RedFLuc model is significantly more invasive and infiltrative than the U87-luc2 model (Fig. 5E).

Additionally, we determined the total number of luciferase-positive cells outside the tumor core with an automated counting algorithm as previously described for other stains (Lagerweij et al. [31]; Schuster et al. [47]). Both methods showed that U251-RedFLuc cells are significantly more invasive and infiltrative than U87-luc2 cells in vivo (Fig. 5 F).

#### Expression of multidrug resistance-related transporters

**Fig. 6:**
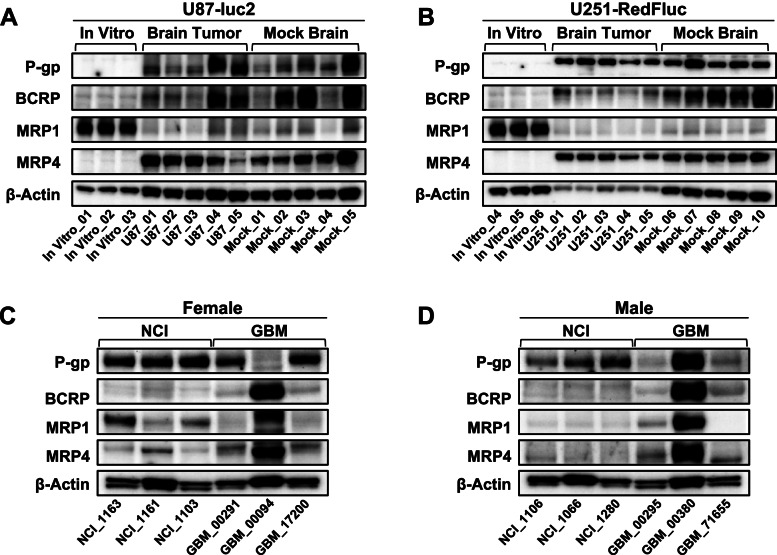
Efflux Transporter Expression in GBM** A** and **B**) Comparison of Protein Expression of the efflux transporters P-gp, BCRP, MRP1, and MRP4 in in vitro and in vivo GBM samples as well as mock control brains. **A** U87-luc2 model (**B**) U251-RedFLuc model. **C** and **D** Protein Expression of the efflux transporters P-gp, BCRP, MRP1, and MRP4 was highly variable among female (**C**) and male (**D**) GBM patients. Data were generated using tissue membrane fraction and normalized to β-actin levels

We analyzed the expression of multidrug resistance-related transporters in both GBM cell lines in vitro and in the GBM mouse models in vivo. In U87-luc2 cells in vitro, expression levels of P-gp, BCRP, and MRP4 were low in the cell membrane fraction (Fig. 6 A, B; Table S4). Membrane fractions of U87-luc2 tumors harvested from mice at the humane endpoint showed significantly increased expression of both P-gp and MRP4 compared to in vitro samples of U87-luc2 cells (Fig. 6 A, B; Table S4; P-gp: ****, *p* < 0.0001; MRP4: *, *p* < 0.05). While BCRP expression increased in brain tumor samples compared to in vitro cell samples, this increase was not statistically significant (Fig. 6 A, B; Table S4**;***p* = 0.13). MRP1 showed the opposite expression profile and was high in in vitro samples but significantly decreased in brain tumor samples (****, *p* < 0.0001; Fig. 6 A, B; Table S4). However, transporter expression was not significantly different in tumor brains compared to mock injected brains (Fig. 6 A, B; Table S4**;***p* = 0.98).

U251-RedFLuc cells showed a similar efflux transporter expression profile as U87-luc2 cells. P-gp, BCRP, and MRP4 expression levels were not detectable in in vitro samples of U251-RedFLuc cells (Fig. 6 C, D; Table S4). Efflux transporter expression was significantly higher in brain tumors compared to in vitro samples (Fig. 6 C, D; Table S4; P-gp: **, *p* = 0.0012; BCRP: **, *p* = 0.0016; MRP4: ***, *p* = 0.0003). As seen in U87-luc2 samples, MRP1 expression showed the opposite expression profile. MRP1 expression was high in in vitro cell samples but was significantly decreased in brain tumor samples (****, *p* < 0.0001; Fig. 6 C, D; Table S4). When comparing tumor samples with mock-injected brain samples, the expression of P-gp (**, *p* = 0.01), MRP1 (*, *p* = 0.043), and MRP4 (**, *p* = 0.0028) was significantly higher in tumor samples (Fig. 6C, D; Table S4). There was no significant difference in BCRP expression between tumor and mock-injected brain samples (*p* = 0.69).

Lastly, we evaluated the expression of P-gp, BCRP, MRP1, and MRP4 in human brain samples from control individuals (CI) who were deceased of GBM-unrelated causes and tumor samples from GBM patients. Patient characteristics are described in Table S2. Expression levels of BCRP, MRP1, and MRP4 were consistently low in both male and female CI samples (Table S5). However, P-gp expression levels in CI samples were higher compared to the other ABC transporters. In general, ABC transporter expression levels in CI brain samples were stable and did not vary much between individuals.

Compared to CI samples, transporter expression was highly variable in human glioblastoma samples of both genders (Table S5). The samples from two patients (#00094, #00380) had much higher expression levels of BCRP, MRP1, and MRP4 than the other patient samples. While patient #00380 also had increased P-gp expression levels, the sample from patient #00094 did not express P-gp. Additionally, patient #71,655 had no detectable expression of MRP1.

To summarize, ABC efflux transporters are expressed at different levels in mouse GBM models and human GBM samples. These data indicate that the expression of multidrug resistance-related transporters at the blood-brain barrier and in tumor cells could be an obstacle to successful treatment in some GBM patients. The U87-luc2 and U251-RedFLuc GBM models express ABC efflux transporters and could model potential treatment effects.

## Discussion

In this study, we established and characterized two human glioblastoma multiforme (GBM) models that recapitulate different aspects of the disease. Specifically, we established two human GBM cell lines (U87-luc2, U251-RedFLuc) that express luciferase enzyme and have similar in vitro characteristics (Fig. 1). Additionally, the viability of both GBM cell lines is dose- and time-dependent after temozolomide and lapatinib exposure (Fig. 2; Table S3).

While both cell lines behave similarly in vitro, the in vivo tumor characteristics recapitulate different aspects of GBM in patients. U87-luc2 cells rapidly grow into large tumors that do not spread from the primary tumor core, and invasion into the contralateral hemisphere only occurs due to the large bulk tumor size (Fig. 4 A; Fig. 5 A, B; Table 2). On the other hand, U251-RedFLuc cells grow slowly and form small tumors. Implantation of more tumor cells is required to achieve significant tumor growth and a similar median survival in U87-luc2 mice (Figs. 3 and 4B; Table 2). However, in mice implanted with U251-RedFLuc cells, luciferase-positive cells are detected throughout the entire brain (Figs. 4B and 5 C, D, G, H). Lastly, we evaluated multidrug resistance-associated ABC transporter expression in both GBM models (Fig. 6; Table S4; Table S5).

Other research groups have previously described several of these tumor characteristics in the U87 and U251 models. However, few studies directly compare the two models, and most include review articles compiling information generated by various laboratories, often with different methods (Candolfi et al. [8]; Radaelli et al. [43]). In addition, most of the previous work was conducted with the parental cell lines, not the luciferase-transduced cell lines. While few publications on the U87-luc2 cell line exist (Phillips et al. [40]; Ranganath et al. [45]; Vandamme et al. [53]), this is the first direct comparison of the U87-luc2 and U251-RedFLuc models. Here, we will discuss our findings in the context of the current literature.

### Luciferase transfection

We observed that luciferase activity in U251-RedFLuc cells was higher than in U87-luc2 cells, independent of luciferase protein expression, even though both GBM cell lines have comparable luciferase protein expression levels (Fig. 1). This observation is most likely due to the different luciferase constructs used when transducing the cell lines. RedFLuc luciferase expressed in U251 cells has a higher photon conversion efficiency than luc2 enzyme expressed in U87 cells. Thus, RedFLuc luciferase more efficiently converts luciferin, resulting in higher bioluminescence levels compared to luc2 luciferase-expressing cells (PerkinElmer, [39]; Branchini et al. [6]; Gil et al. [20]; Liang et al. [34]), which results in higher luciferase activity in U251-RedFLuc cells compared to U87-luc2 cells.

### *In Vitro* cytotoxicity

Temozolomide is the standard of care treatment for GBM, yet many patients are treatment-resistant (Hegi et al. [26]). The most common mechanism of temozolomide resistance is based on O6-methylguanine-DNA methyltransferase (MGMT)-mediated demethylation, which removes methyl groups from DNA alkylated by agents like temozolomide (Hegi et al. [26]). MGMT is expressed in approximately 55% of GBM patients, rendering temozolomide and other DNA alkylating agents ineffective. Since neither U87 nor U251 cells express MGMT protein, we anticipated that temozolomide would decrease the viability of U251 and U87 cells in a dose-dependent manner (Trevisan et al. [52]; Yi et al. [60, 61]). However, we found that the cell viability of U87-luc2 cells was minimally decreased 72 h after exposure to 500 µM TMZ; the cell viability of U251-RedFluc cells was minimally decreased after 48 and 72 h after exposure to 500 µM TMZ (Fig. 2 A-C). Based on these data the IC50 for TMZ is > 500 µM for both cell lines. In contrast, lapatinib significantly decreased the viability in both GBM cell lines in a time- and dose-dependent manner (Fig. 2; Table S3). Thus, U87-luc2 and the U251-Fluc cells appear to be significantly less sensitive to temozolomide compared to lapatinib. It is noteworthy that both U87 and U251 cells express wild-type epidermal growth factor receptor (EGFR), which is the target of lapatinib (Bigner et al. [4]; Wachsberger et al. [55]). Our data suggest that lapatinib could potentially be a beneficial anticancer drug for the treatment of GBM. However, limited brain uptake across the blood-brain barrier diminishes the efficacy of lapatinib in GBM treatment (Polli et al. [41]; Thiessen et al. [50]).

### *In Vivo* tumor characteristics

As shown above, both cell lines behave similarly in vitro. However, their in vivo tumor characteristics such as tumor growth, tumor volume, tumor cell invasiveness, and ABC transporter expression levels recapitulate different aspects of glioblastoma in patients.

### Tumor growth and volume

In our MRI studies, we used a T2-RARE sequence to determine anatomical features and edema combined with a standard T1 scan to evaluate contrast enhancement and tumor volume. We observed that U87-luc2 cells rapidly grew into large tumors. After intracranial injection of 150,000 cells, U87-luc2 tumors doubled in size approximately every 7 days until they reached 120 to 180 mm^3^ around the time of median survival (41 days; Figs. 3 and 4; Tables 1 and 2). U87-luc2 tumors were well-demarcated and easily identifiable on both T1 and T2 scans (Fig. 4 A). Additionally, the large tumors allowed contrast agents to cross the blood-tumor barrier, as indicated by heterogeneous enhancement on T1 MRI scans (Fig. 4 A; Candolfi et al. [8]; de Vries [11]; de Vries et al. [12]; Radaelli et al. [43]). However, these main histopathological and imaging characteristics of U87 tumors are not representative of most glioblastoma patients (Radaelli et al. [43]). Therefore, extrapolation from this preclinical model to the clinic should be handled with caution.

Unlike the U87 model, U251 cells recapitulate many of the main glioblastoma characteristics observed in patients. U251-RedFLuc cells grew slowly and formed small tumors. For more significant tumor growth and a median survival similar to that in U87-luc2 mice, more tumor cells need to be implanted. Consistent with this, we observed in mice injected with 1,000,000 U251-RedFLuc cells that the average tumor doubling time was 14 days, and the tumor cores grew to about 50 mm^3^ in volume by the time of median survival (Figs. 3 and 4; Tables 1 and 2). U251-RedFLuc tumors appeared to be poorly demarcated with limited contrast enhancement on T1 scans. However, tumors were often enhanced around the tumor rim on T2 scans, indicating edema (Fig. 4B; (Radaelli et al. [43]). These features observed in the U251-RedFLuc model recapitulate important tumor characteristics seen in many glioblastoma patients. Interestingly, even though U251-RedFLuc tumors remained relatively small, median survival of U251-RedFLuc mice was 35 days (Fig. 3; Table 1). This discrepancy between tumor volume and animal survival is most likely due to the invasiveness of U251-RedFLuc cells. To test this hypothesis, we quantified the invasiveness of both GBM models.

### Tumor cell invasiveness

Previous publications describe the invasiveness of GBM cells by staining for human proteins, such as human leukocyte antigen (Candolfi et al. [8]; Radaelli et al. [43]). However, staining for human proteins limits this approach to human glioblastoma models in immunocompromised mice. Additionally, cell invasion was quantified with subjective scoring methods, while objective quantitative analysis of GBM cell infiltration in vivo has only recently been described (Lagerweij et al. [31]; Schuster et al. [47]). Here, we adapted these two protocols to automatically quantify luciferase-positive GBM cells outside of the tumor core (Fig. 5E, F, G, H), which will allow the analysis of GBM invasiveness in both human and murine GBM models.

Using this new anti-luciferase staining method, we confirmed that U251-RedFLuc cells are more invasive than U87-luc2 cells (Fig. 5). Indeed, in mice implanted with U251-RedFLuc cells, luciferase-positive cells are detected throughout the entire brain (Fig. 5G, H; Candolfi et al. [8]; Radaelli et al. [43]). On the other hand, luciferase-positive U87-luc2 cells do not spread from the primary tumor core, and invasion into the contralateral hemisphere only occurs due to the large size of the bulk tumor (Fig. 5 A, B). This is consistent with studies showing that, unlike patient tumors, U87 tumors have a non-infiltrative growth pattern and are unlikely to recur after resection (Candolfi et al. [8]; de Vries [11]; de Vries et al. [12]; Radaelli et al. [43]).

Clinically, GBM can be distinguished into four molecular subtypes. Based on the reported genomic variations, both U87-luc2 and U251-RedFLuc cells can be classified as proneural GBM (Verhaak et al. [54]; Brennan et al. [7]). Therefore, GBM subtype does not appear to be a driver of the different growth patterns. Further research is needed to identify the cause for the different in vivo growth patterns of U87-luc2 and U251-RedFLuc cells.

### ABC transporter expression

Lastly, we evaluated multidrug resistance-associated ABC transporter expression in both GBM models. Generally, ABC transporter expression followed two different trends. First, P-gp, BCRP, and MRP4 expression were low in in vitro samples from both cell lines. While transporter expression was higher in in vivo vs. in vitro samples, it was not significantly different from mock-injected control brains (Fig. 6; Table S4). Second, MRP1 expression was high in vitro and compared to that lower in in vivo brain tumor samples from both models (Fig. 6; Table S4).

Previously published reports confirmed the ABC transporter expression patterns in U87 and U251 cells in vitro. P-gp and BCRP expression in both U87 and U251 cells were low (Doganlar et al. [13]; Wang et al. [57]; Zhang et al. 62), while MRP1 was expressed at high levels in both cell lines (Li et al. [33]; Yao and Zhang [59]). However, no information was available on MRP4 protein expression in U87 and U251 cells in vitro. Here, we show that MRP4 protein levels are low in U87 and U251 cells (Fig. 6; Table S4). Additionally, we show that ABC transporter expression levels in cell culture in vitro differ from those in the GBM model in vivo after intracranial cell implantation. However, transporter expression levels in GBM samples were not significantly different from those in brain samples from mock-injected control mice (Fig. 6; Table S4). Based on these observations it is likely that cells in the tumor microenvironment in the brain, such as astrocytes and brain vascular endothelial cells, also express ABC transporters (Talele et al. [49]). Future research should discern the mechanisms that drive changes in ABC transporter expression and the impact of the tumor microenvironment in vivo. Notably, changes in transporter expression in tumor cells compared to the blood-tumor barrier need to be carefully evaluated. Future studies should determine the impact of different therapeutic strategies on the expression of ABC transporters at the blood-brain barrier as well as in glioblastoma cells.

## Conclusions

To our knowledge, this is the first direct comparison of the luciferase-expressing human GBM models U87-luc2 and U251-RedFLuc. We provide a detailed overview of in vitro and in vivo characteristics of these GBM models, including tumor growth, median survival, tumor imaging characteristics, and invasiveness. Here, we also present a new method to quantify GBM invasiveness in vivo using anti-luciferase IHC staining.

## Supplementary Information

**Additional file 1: Table S1. **GBM Implantation Protocols. **Table S2. **Demographics of GBM patients. **Table S3. **Cytotoxicity of Temozolomide (TMZ) and Lapatinib (LAP) in GBM cell lines. **Table S4. **Transporter Expression in Mouse GBM Models. **Table S5. **Transporter Expression in Human GBM Samples.

**Additional file 2: Figure S1.** Luciferase Standard Curve Dot Blot.

**Additional file 3: Figure S2. **Cytotoxicity of DMSO in GBM cells in vitro Cytotoxicity of DMSO in GBM cells in vitro.

## Data Availability

The datasets used and analyzed during the current study are available from the corresponding author on reasonable request.

## Abbreviations

GBM: Glioblastoma
TMZ: Temozolomide
LAP: Lapatinib
MRI: Magnetic Resonance Imaging
P-gp: P-glycoprotein (ABCB1)
BCRP: Breast Cancer Resistance Protein (ABCG2)
MRP1: Multidrug-resistance associated protein 1 (ABCC1)
MRP4: Multidrug-resistance associated protein 4 (ABCC4)
