# Supplementary material for: Characterization and comparison of human glioblastoma models
Source: BMC Cancer. 2022 Aug 3;22:844. doi: 10.1186/s12885-022-09910-9 (PMC9347152; doi:10.1186/s12885-022-09910-9)
Supplement: Supplementary file 1 — Additional file 1: Table S1. GBM Implantation Protocols. Table S2. Demographics of GBM patients. Table S3. Cytotoxicity of Temozolomide (TMZ) and Lapatinib (LAP) in GBM cell lines. Table S4. Transporter Expression in Mouse GBM Models. Table S5. Transporter Expression in Human GBM Samples. [file 12885_2022_9910_MOESM1_ESM.docx]

**Characterization and Comparison of Human Glioblastoma Models**

**Abbreviated Title:** GBM Model Comparison

**Julia A. Schulz^1^, Louis T. Rodgers^1^, Richard J. Kryscio^2,3^, Anika M.S. Hartz^2,4^, Björn Bauer^1,5,*^**

^1^ Pharmaceutical Sciences, College of Pharmacy, University of Kentucky

^2^ Sanders-Brown Center on Aging, University of Kentucky

^3^ Statistics, College of Arts and Sciences, University of Kentucky, Lexington, KY, 40536, USA

^4^ Pharmacology and Nutritional Sciences, College of Medicine, University of Kentucky

^5^ Drug Discovery, Delivery and Translational Therapeutics Track, Markey Cancer Center, College of Medicine, University of Kentucky

***Corresponding Author:** Björn Bauer, PhD

University of Kentucky

College of Pharmacy

Department of Pharmaceutical Sciences

333 Sanders-Brown Center on Aging

800 S Limestone

Lexington, KY 40536-0230

Email: bjoern.bauer@uky.edu

**SUPPLEMENTAL DATA**

**Table S1. GBM Implantation Protocols.**

| **Cell Line** | **Total Cell #, Volume [μl]** | **Cell Concentration [cells/μl]** | **Injection Rate [μl/min] & Time** |
| --- | --- | --- | --- |
| **U87-luc2** | 150,000 (3 μl) | 50,000 | 1 (3 min) |
| **U251-RedFLuc** | 150,000 (3 μl)  250,000 (5 μl)  500,000 (5 μl)  1,000,000 (10 μl) | 50,000  50,000  100,000  100,000 | 1 (3 min)  1 (5 min)  1 (5 min)  2 (5 min) |

**Table S2. Demographics of GBM patients.**

| **Sample ID** | **Origin** | **Diagnosis** | **Gender** | **Age** | **Ethnicity** |
| --- | --- | --- | --- | --- | --- |
| 17200 | MCC | GBM | Female | 69 | Caucasian |
| 71655 | MCC | GBM | Male | 56 | Caucasian |
| MAD13-00295 T2-5 | CHTN | GBM | Male | 49 | Caucasian |
| MAD13-00094 T1-9 | CHTN | GBM | Female | 44 | Caucasian |
| MAD13-00291 T1-5 | CHTN | GBM | Female | 53 | Caucasian |
| MAD14-00380 T1-3 | CHTN | GBM | Male | 71 | Caucasian |
| 1103 | UK | CI | Female | 76 | Caucasian |
| 1161 | UK | CI | Female | 84 | Caucasian |
| 1163 | UK | CI | Female | 84 | Caucasian |
| 1280 | UK | CI | Male | 86 | Caucasian |
| 1066 | UK | CI | Male | 81 | Caucasian |
| 1106 | UK | CI | Male | 79 | Caucasian |

**Table S3. Cytotoxicity of Temozolomide (TMZ) and Lapatinib (LAP) in GBM cell lines.**

| **IC50 [µM]** | **24h** | | **48h** | | **72h** | |
| --- | --- | --- | --- | --- | --- | --- |
| **Cell Line** | **TMZ** | **LAP** | **TMZ** | **LAP** | **TMZ** | **LAP** |
| **U87-luc2** | > 500 µM  (4,395 µM*) | 238 µM | > 500 µM  (2,253 µM*) | 106.4 µM | > 500 µM  (1,150 µM*) | 4.78 µM |
| **U251-FLuc** | > 500 µM  (4,046 µM*) | 227.9 µM | > 500 µM  (1,337 µM*) | 119.3 µM | > 500 µM  (1,981 µM*) | 18.8 µM |

IC50 values for Temozolomide (TMZ) and Lapatinib (LAP) in U87-luc2 and U251-RedFLuc cells. * Extrapolated IC50 values. Statistics: unpaired *t*-test ****, *p*< 0.0001 (n=4, 3 technical replicates).

**Table S4. Transporter Expression in Mouse GBM Models.**

| **Protein** | **P-gp** | **Bcrp** | **Mrp1** | **Mrp4** |
| --- | --- | --- | --- | --- |
| **U87-luc2 *in vitro*** | 0.01 ± 0.01 | 0.59 ± 0.03 | 5.12 ± 0.61 | 0.18 ± 0.02 |
| **U87-luc2 *in vivo*** | 1.24 ± 0.09 **** | 0.94 ± 0.06  ns | 0.68 ± 0.06 **** | 1.09 ± 0.29  * |
| **Control Brain** | 1.10 ± 0.10 **** | 1.14 ± 0.13  * | 0.66 ± 0.07 **** | 0.95 ± 0.14  ns |
| **U251-RedFLuc *in vitro*** | 0.01 ± 0.003 | 0.07 ± 0.03 | 1.46 ± 0.04 | 0.27 ± 0.02 |
| **U251-RedFLuc *in vivo*** | 2.48 ± 0.43  ** | 1.09± 0.18  ** | 0.50 ± 0.08 **** | 2.36 ± 0.33 *** |
| **Control Brain** | 0.93 ± 0.17 | 0.94 ± 0.08  ** | 0.26 ± 0.03 **** | 1.00 ± 0.05  ns |

Data were normalized to β-actin levels. Statistics: Ordinary One-Way ANOVA with Tukey’s multiple comparisons test (results compared to *in vitro*) **, *p*<0.01; ***, *p*<0.001; ****, *p*<0.0001; (*in vitro* n=3, *in vivo* n=5, Control n=5; 2 technical replicates).

**Table S5. Transporter Expression in Human GBM Samples**

|  | | | **P-gp** | **BCRP** | **MRP1** | **MRP4** |
| --- | --- | --- | --- | --- | --- | --- |
| **Female** | **CI** | **1163** | 2.59 | 0.28 | 1.71 | 0.40 |
|  |  | **1161** | 1.26 | 0.32 | 0.43 | 0.36 |
|  |  | **1103** | 3.55 | 0.28 | 0.125 | 0.33 |
|  |  | **Average** | 2.47 ± 0.67 | 0.30 ± 0.01 | 1.13 ± 0.37 | 0.36 ± 0.02 |
|  | **GBM** | **00291** | 2.34 | 0.49 | 0.91 | 1.02 |
|  |  | **00094** | 0.49 | 1.26 | 1.16 | 1.13 |
|  |  | **17200** | 2.21 | 0.50 | 0.74 | 0.85 |
|  |  | **Average** | 1.68 ± 0.59, ns | 0.75 ± 0.26, ns | 0.93 ± 0.12, ns | 1.00 ± 0.08, ** |
| **Male** | **CI** | **1106** | 1.87 | 1.9 | 0.5 | 2.1 |
|  |  | **1066** | 2.91 | 2.7 | 0.8 | 2.2 |
|  |  | **1280** | 2.88 | 2.3 | 0.7 | 1.6 |
|  |  | **Average** | 2.55 ± 0.34 | 2.32 ± 0.22 | 0.66 ± 0.07 | 1.97 ± 0.17 |
|  | **GBM** | **00295** | 0.27 | 0.4 | 0.3 | 0.7 |
|  |  | **00380** | 0.92 | 1.1 | 1.2 | 1.2 |
|  |  | **71655** | 5.06 | 7.4 | 0.4 | 7.1 |
|  |  | **Average** | 2.09 ± 1.50, ns | 2.97 ± 2.21, ns | 0.65 ± 0.27, ns | 3.02 ± 2.06, ns |

Data were normalized to β-actin levels. Statistics: Unpaired t-test (GBM vs. CI) **, *p*<0.01; (n=3/group; 2 technical replicates).
