# Supplementary material for: Characterization and comparison of human glioblastoma models
Source: BMC Cancer. 2022 Aug 3;22:844. doi: 10.1186/s12885-022-09910-9 (PMC9347152; doi:10.1186/s12885-022-09910-9)
Supplement: Supplementary file 2 — Additional file 2: Figure S1. Luciferase Standard Curve Dot Blot. [file 12885_2022_9910_MOESM2_ESM.pdf]

Figure S1. Luciferase Standard Curve Dot Blot.

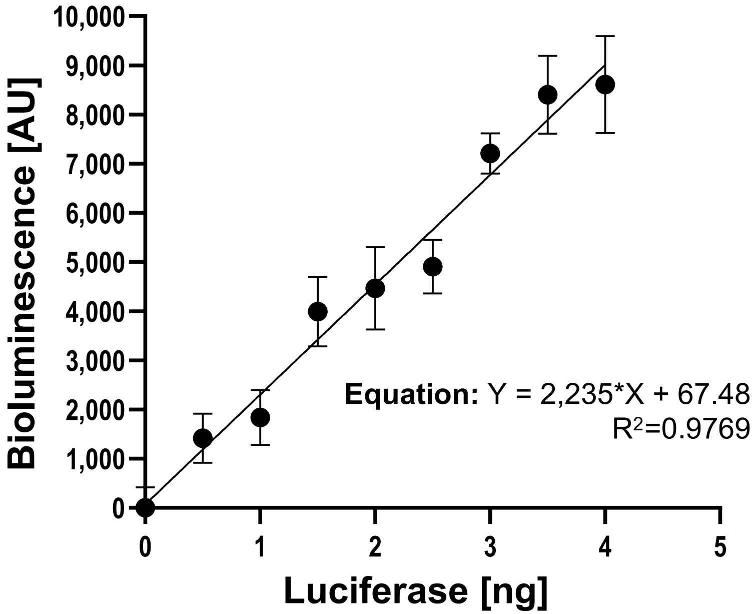

Figure S1. Representative standard curve for dot blot quantification of luciferase expression. Recombinant luciferase (ab100961, Abcam, Cambridge, MA, USA) was diluted to 0.0025-0.02 ng/µl (0.5 – 4 ng total protein content per well). 200 µl of each solution was loaded on Amersham Protran Nitrocellulose Membranes (115-125 µg IgG/cm², GE Healthcare, Chicago, IL, USA) using a vacuum pump. Membranes were processed, and images were acquired as described under Western blotting. Luciferase protein was detected with the Anti-Luciferase antibody (ab187340, 0.25 µg/µl). Images were analyzed with ImageLab 6.1.0 (BioRad, Hercules, CA, USA), and the standard curve was determined with GraphPad Prism® (version 9). The Luciferase amount was quantified and calculated based on the respective standard curves.
