# Supplementary material for: Characterization and comparison of human glioblastoma models
Source: BMC Cancer. 2022 Aug 3;22:844. doi: 10.1186/s12885-022-09910-9 (PMC9347152; doi:10.1186/s12885-022-09910-9)
Supplement: Supplementary file 3 — Additional file 3: Figure S2. Cytotoxicity of DMSO in GBM cells in vitro Cytotoxicity of DMSO in GBM cells in vitro. [file 12885_2022_9910_MOESM3_ESM.pdf]

# Figure S2. Cytotoxicity of DMSO in GBM cells *in vitro*.

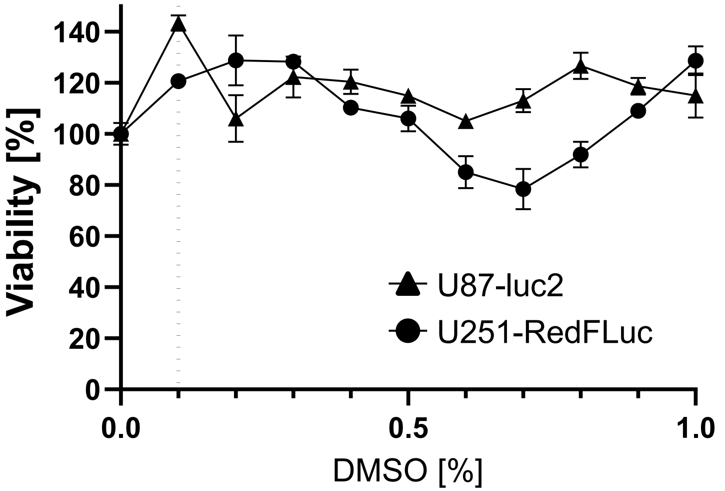


**Figure S2.** Exposure to DMSO for 24h did not affect cell viability in U87-luc2 or U251-RedFLuc cells at DMSO concentrations up to 1%; DMSO concentration used for cytotoxicity experiments (0.1%; dotted line). Statistics: Ordinary One-Way ANOVA (results compared to 0% DMSO) ns, $p > 0.05$ (n=3; Mean ± SEM).
